# A Rare Case of Cocklebur Foreign Body in the Larynx

**DOI:** 10.7759/cureus.72327

**Published:** 2024-10-24

**Authors:** Mitsuki Toda, Kosuke Akiyama, Hiroshi Hoshikawa

**Affiliations:** 1 Otolaryngology - Head and Neck Surgery, Kagawa University, Takamatsu, JPN

**Keywords:** cocklebur, foreign body, larynx, plant foreign matter, xanthium strumarium

## Abstract

Plant foreign matter in the larynx is rare. Only three cases of a cocklebur foreign body in the larynx have been previously reported. A 55-year-old man accidentally swallowed cocklebur fruit. The cocklebur fruit attached to the front of the glottis. The patient had a strong sore throat and a feeling of a foreign body. Considering that removal was difficult at the nearest emergency outpatient clinic because of the strong adhesion and the patient’s cough reflex, general anesthesia was induced by intravenous anesthetics and muscle relaxants, the larynx was expanded with McGrath's laryngeal forceps, and the foreign body was removed using Magill forceps. Granulation and erosion of the larynx persisted. Two weeks later, the laryngeal mucosa normalized, and the patient was cured completely. The International Liaison Committee on Resuscitation International Consensus recently recommended the use of Magill forceps for airway foreign body removal in 2020. In this case, Magill forceps were able to grasp the entire fruit without leaving hard spines and were useful in removing the foreign body.

## Introduction

Foreign bodies in the larynx are relatively rare due to the larynx's cough reflex, which acts as a defense mechanism by expelling small foreign bodies. If the glottis is blocked, asphyxiation may occur, and emergency airway management is necessary. Most foreign bodies are related to food [1]. Inanimate foreign bodies are rare, and plant foreign bodies are rarer. Herein, we describe an extremely rare case of a cocklebur (Xanthium strumarium) foreign body in the larynx of a healthy middle-aged man. Verbal consent was obtained from the patient in accordance with the hospital's regulations.

## Case presentation

During farm work, a 55-year-old male tried to remove a cocklebur fruit from his glove using his mouth and accidentally swallowed it. He had a sore throat and hoarseness immediately. He visited the nearest emergency outpatient clinic. A gastroenterologist confirmed the foreign body in his larynx by upper gastrointestinal endoscopy and attempted to remove it with biopsy forceps. However, it was difficult to remove due to its strong snag and the patient’s cough reflex. Therefore, the patient was referred to our hospital on the same day. A brownish foreign body was found in front of the glottis, surrounded by secretions. The vocal fold gap was preserved, and the patient was able to speak and had no dyspnea. His vital signs were mostly normal: body temperature 36.8°C, pulse rate 58 times/min, and saturation of percutaneous oxygen 98%. However, his blood pressure was elevated at 156/108 mmHg. There was salivary retention in both piriform sinus, and mild edematous changes were observed from the left laryngeal region to the laryngopharyngeal folds (Video 1).

**Video 1:** Laryngoscopy findings There is salivary retention in both piriform sinus. Mild edematous changes are observed from the left laryngeal region to the laryngopharyngeal folds.

**Figure 1:**
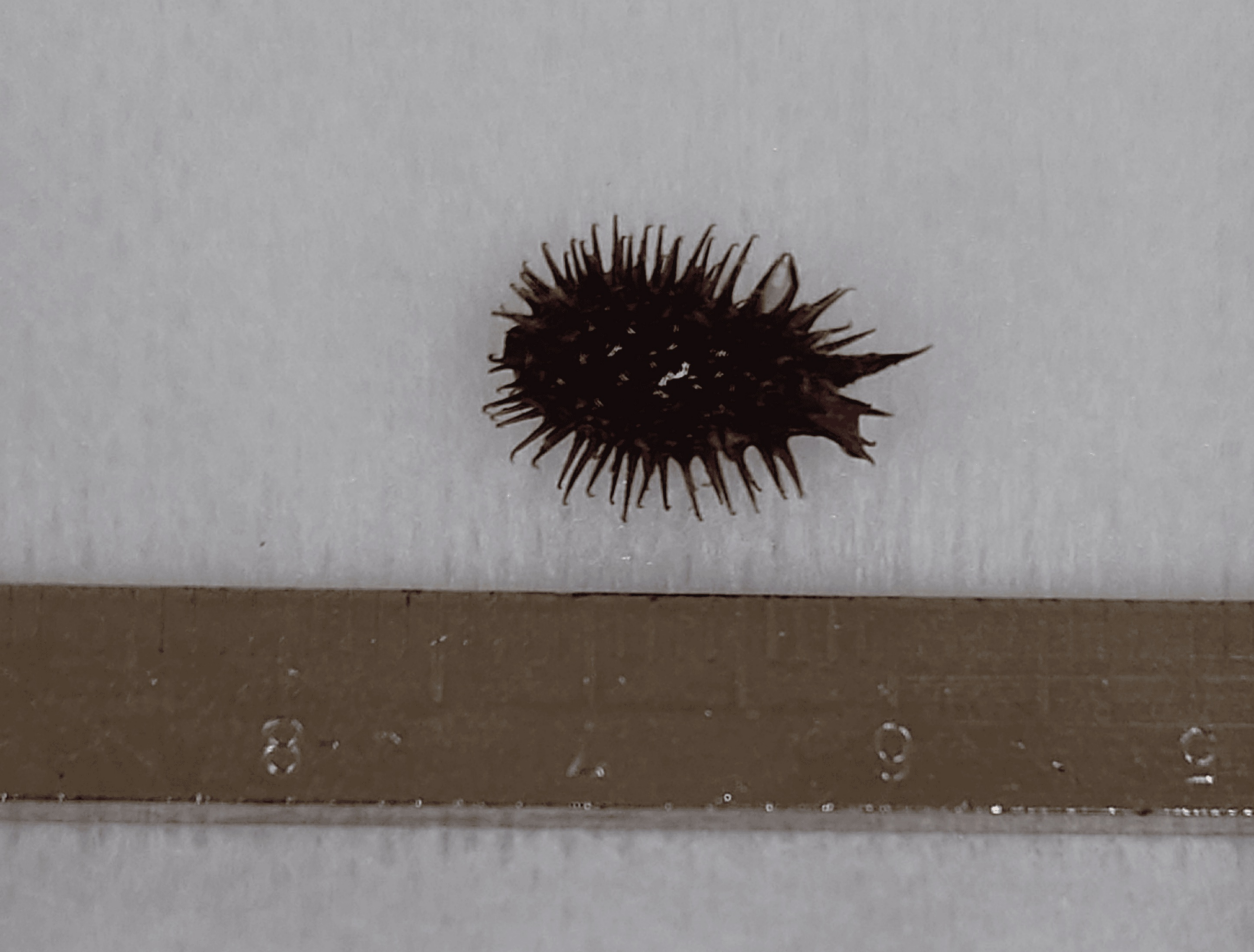
The foreign body A cocklebur fruit is 12×7 mm in size and has numerous hard spines.

**Figure 2:**
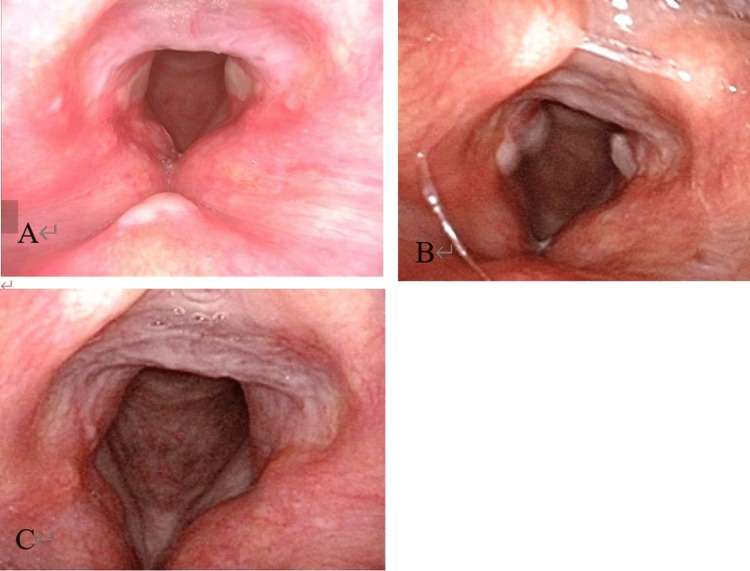
Laryngoscopy findings after removal of the foreign body A: one day after removal (the laryngeal mucosa and bilateral vocal fold are erosive); B: ten days after removal (there are granulations on bilateral vocal fold processes); C: twenty-four days after removal (the granulations disappeared and the mucosa became epithelialized)

At the time of the initial examination, we attempted laryngeal foreign body removal. Considering that removal was difficult at the nearest emergency outpatient clinic because of the strong adhesion and the patient’s cough reflex, we decided to remove the foreign body under general anesthesia rather than inhalation or surface anesthesia. In this case, extraction and airway management could be performed safely in the emergency room under the backup of an emergency physician with sufficient experience in anesthesia and intubation management. The patient did not have severe airway narrowing at the time of removal, and airway management was not expected to be difficult. However, if the size of the foreign body and airway status were more severe, it may be necessary to perform the procedure in the operating room under the supervision of an anesthesiologist to enhance safety. After induction of general anesthesia with administration of intravenous anesthetics (propofol 120 mg, fentanyl 0.25 mg) and muscle relaxants (rocuronium 50 mg) by the emergency physician, the larynx was expanded with McGrath's laryngeal forceps, and the foreign body was removed using Magill forceps in the emergency room. The foreign body was a 12 x 7 mm-sized cocklebur fruit with numerous hard spines (Figure 1). The laryngeal mucosa was erosive after removal (Figure 2A). The patient was intubated. To improve the laryngeal edema, 40 mg of methylprednisolone was administered intravenously every six hours (four times total: 160 mg). The next day after removal, laryngoscopy confirmed improvement of edema in the left laryngeal region, and a cuff leak test was performed and extubation was performed. He was discharged four days after surgery. When the patient was reexamined seven days after discharge, granulations were seen on the bilateral vocal fold processes. We administered tranexamic acid to be taken orally for two weeks. The granulations disappeared and the mucosa became epithelialized (Figures 2B, 2C).

## Discussion

Cocklebur (X. strumarium) is an annual weed of the Asteraceae family that grows wild in forests, mountain meadows, and wastelands. Its fruits are 1-2 cm long, oval-shaped, and covered with hooked spines. In Japan, it grows wild over a wide area and is a common plant. It is known by the common name "snagging insect" because it often snags clothing. Airway foreign bodies, including those in the larynx, are most common in infants, who are prone to placing objects in their mouths. However, they are also found in the elderly, indicating a bimodal distribution. The most common types of foreign bodies in infants are small foods, such as peanuts, and small toys. On the other hand, fish bones, dentures, and press-through packages are more common in adults. To the best of our knowledge, only three cases of bronchial foreign bodies caused by cocklebur fruit have been reported in the English literature, all of which were removed by direct laryngoscopy and bronchoscopy (Table 1) [2,3,4].

**Table 1:** Time to removal and the method of extraction for previously reported cases The present case is case 4.

	Age	Sex	Time to removal	Extraction method
Case 1 [2]	8 months	Male	4 days	Direct laryngoscopy. Type of forceps not reported.
Case 2 [3]	50 years	Male	1 year	Bronchoscopy. Type of forceps not reported.
Case 3 [4]	7 years	Male	2 days	Bronchoscopy and crocodile clamp.
Case 4	55 years	Male	7 hours	McGrath's laryngeal forceps and Magill forceps.

**Figure 3:**
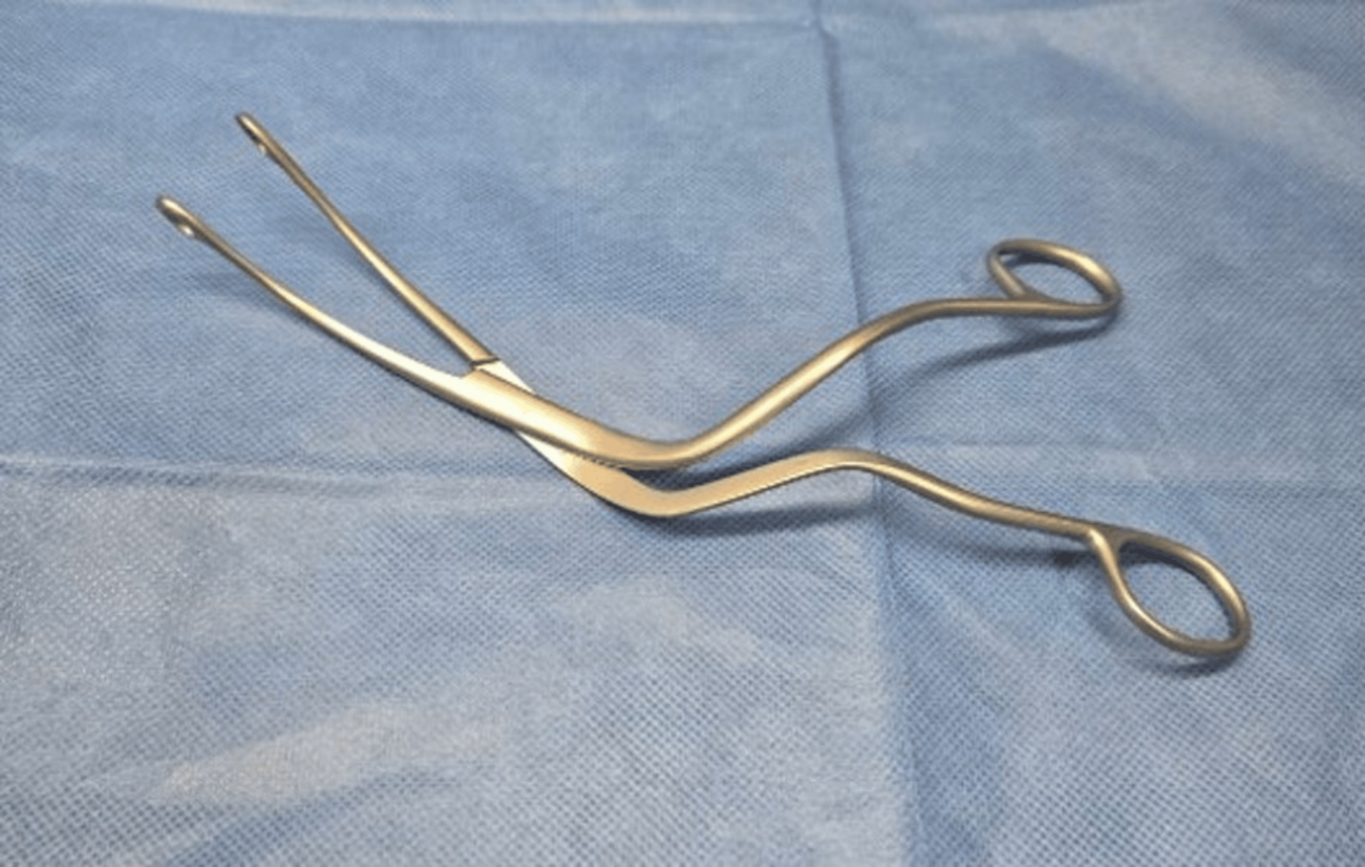
Magill forceps

In the present case, the previous physician failed to remove the foreign body due to the patient’s strong cough reflex and the large size of the foreign body. Biopsy forceps may be useful for the removal of long, thin foreign bodies, such as fish bones, but should not be used for large foreign bodies that cannot be grasped in their entirety. The patient expressed anxiety about treatment because the procedure was highly invasive; therefore, we selected to remove the foreign body using muscle relaxants under sedation. Further irritation should be avoided due to the development of laryngeal edema. For the removal of the cocklebur foreign body, it was necessary to grasp the fruit without leaving any spines; therefore, laryngeal ultrasound or CT scan may have been useful to collect supplemental data on the location of the foreign body. We used Magill forceps to grasp the entire fruit and remove the foreign body. Magill forceps are generally used by anesthesiologists to guide the intubation tube toward the glottis when performing nasal intubation; however, the International Liaison Committee on Resuscitation International Consensus recently recommended the use of Magill forceps for airway foreign body removal in 2020 [5]. The characteristics of the forceps make them easy to use, allowing for a firm grasp with minimal force (Figure 3). This method facilitated the safe and reliable removal of the foreign body. In the present case, laryngeal granulation occurred after removal of the foreign body; thus, close observation and nebulizer therapy were necessary. The foreign body was present in the larynx for about five hours and was thought to be the cause of the prolonged time required for the granulation to resolve. Our findings suggest the need to consider reliable extraction methods for complex and spiky foreign bodies, such as cocklebur fruit, which may cause damage to surrounding tissues during removal.

## Conclusions

We encountered an extremely rare case of a cocklebur foreign body in the larynx, positioned anteriorly within the laryngeal chamber. Considering that removal was difficult at the nearest emergency outpatient clinic because of the strong adhesion and the patient’s cough reflex, the larynx was expanded with McGrath’s laryngeal forceps, and the foreign body was removed using Magill forceps under general anesthesia. A laryngeal foreign body should be diagnosed and treated early, before it falls out. In addition, instruments that can grasp the entire foreign body, such as Magill forceps, should be used to ensure that there are no residuals. 
